# Correction: Cervical cancer screening utilization and predictors among eligible women in Ethiopia: A systematic review and meta-analysis

**DOI:** 10.1371/journal.pone.0294799

**Published:** 2023-11-16

**Authors:** Melaku Desta, Temesgen Getaneh, Bewuket Yeserah, Yichalem Worku, Tewodros Eshete, Molla Yigzaw Birhanu, Getachew Mullu Kassa, Fentahun Adane, Yordanos Gizachew Yeshitila

The third author’s name is spelled incorrectly. The correct name is: Bewket Yeserah Aynalem. The correct citation is: Desta M, Getaneh T, Aynalem BY, Worku Y, Eshete T. Birhanu MY, et al. (2021) Cervical cancer screening utilization and predictors among eligible women in Ethiopia: A systematic review and meta-analysis. PloS ONE 16(11): e0259339. https://doi.org/10.1371/journal.pone.0259339
