# Supplementary figures and images for: Cervical cancer screening utilization and predictors among eligible women in Ethiopia: A systematic review and meta-analysis
Source: PLoS One. 2021 Nov 4;16(11):e0259339. doi: 10.1371/journal.pone.0259339 (PMC8568159; doi:10.1371/journal.pone.0259339)

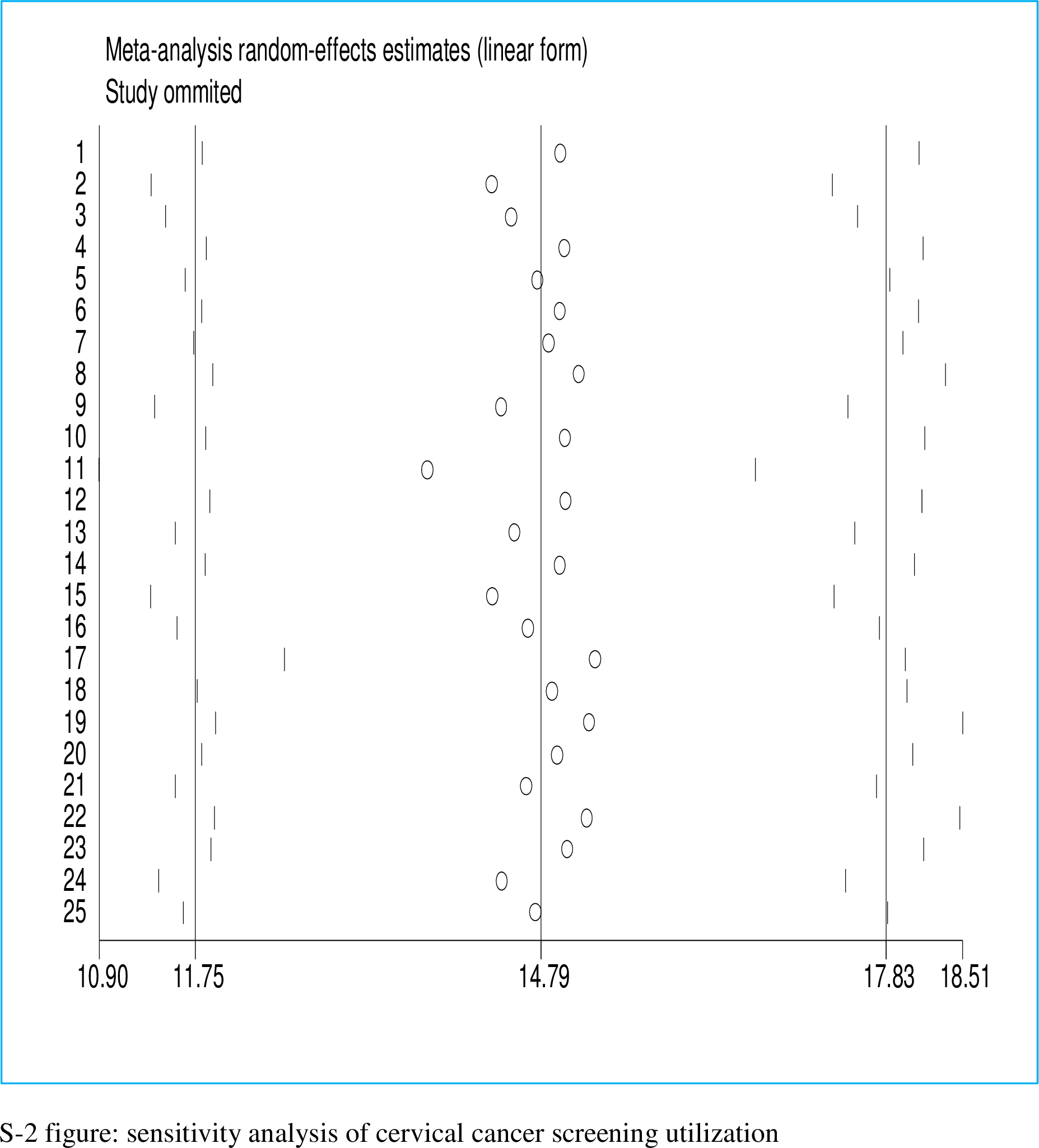

Supplement: S1 Fig — (TIF) [file pone.0259339.s001.tif]

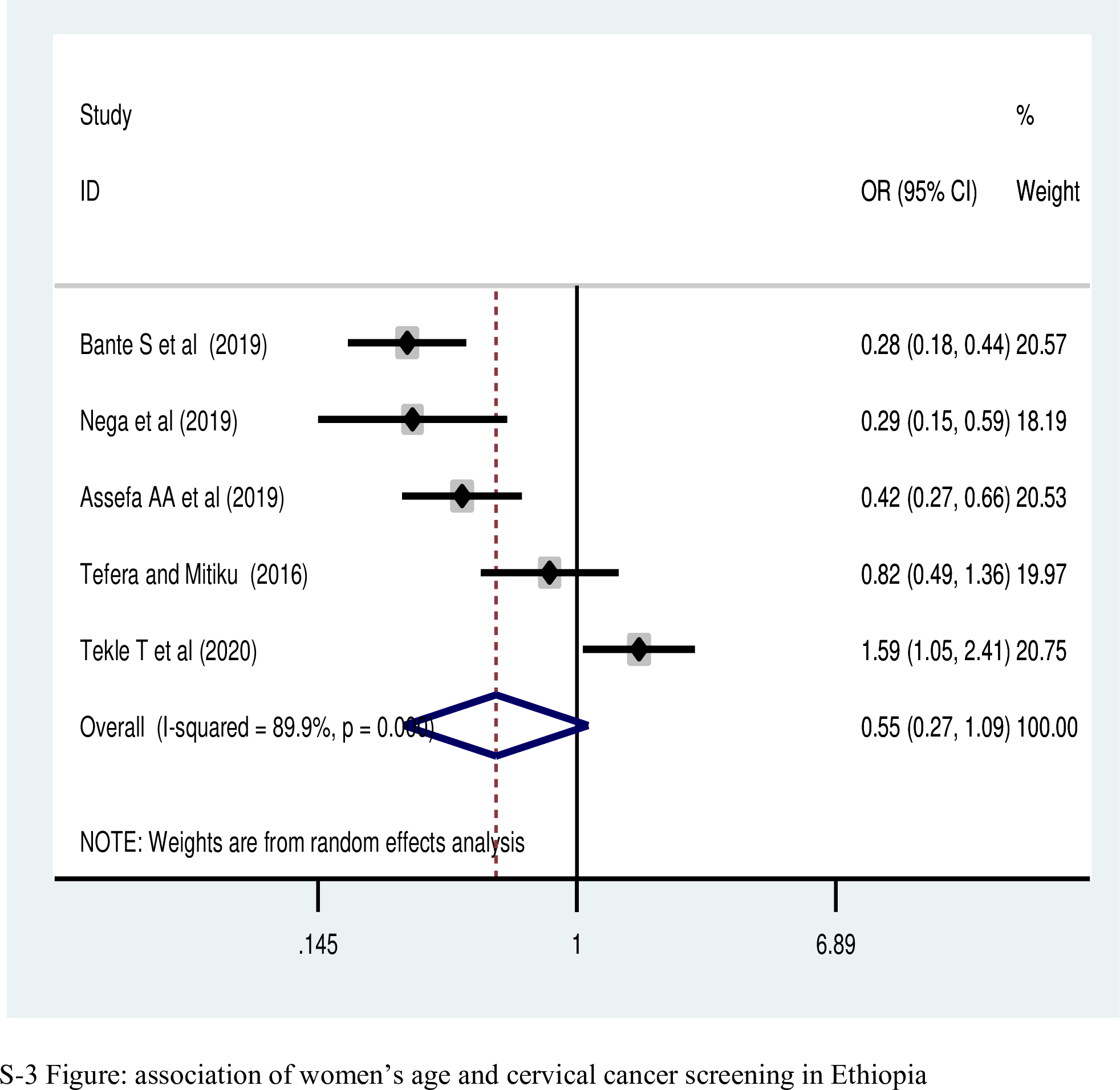

Supplement: S2 Fig — (TIF) [file pone.0259339.s002.tif]

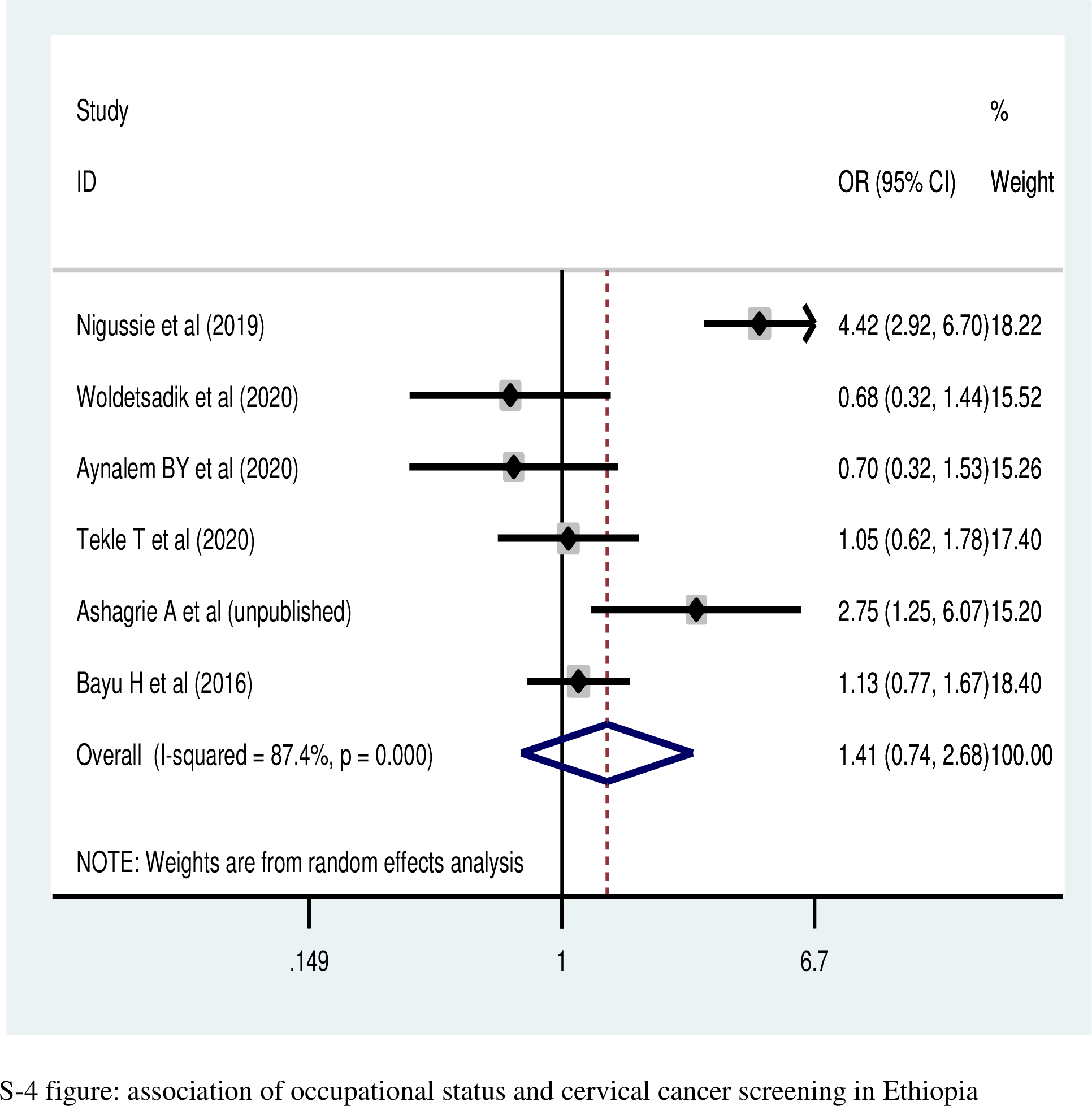

Supplement: S3 Fig — (TIF) [file pone.0259339.s003.tif]
