# Supplementary material for: Cervical cancer screening utilization and predictors among eligible women in Ethiopia: A systematic review and meta-analysis
Source: PLoS One. 2021 Nov 4;16(11):e0259339. doi: 10.1371/journal.pone.0259339 (PMC8568159; doi:10.1371/journal.pone.0259339)
Supplement: S2 Table — (DOCX) [file pone.0259339.s005.docx]

**S2 Table: included studies for the barriers of cervical cancer screening**

| authors | Healthy | Don’t know service site | fear of Procedure | Long waiting | Lack of information | embarrassment | sample |
| --- | --- | --- | --- | --- | --- | --- | --- |
| Bayu et al | 72.7 | -α | 60.1 | 41.5 | -α | 45.6 | 1186 |
| Ashagrie et al | 22.2 | 14.1 | 1.6 | 0.3 | -α | -α | 318 |
| Tekle et al | -α | -α | 10.5 | 51.4 | 69.8 | 26 | 520 |
| Nigussie et al | 66.2 | -α | 3.1 | -α | 19.9 | 1.2 | 737 |
| Shiferaw | 36.5 | 20.1 | 5.3 | -α | 9.6 | 5.5 | 598 |
| Getachew S | 57.6 | -α | 5.4 | -α | 56.3 | 6.7 | 520 |
| Bante SA | 54 | -α | 11.2 | 16.6 | 35.2 | 1.7 | 577 |
| Aweke et al | 35.8 | 2.1 | -α | -α | -α | 0.3 | 583 |
| Assefa AA et al | 34.1 | 6.3 | 16.1 | -α | 36.6 | -α | 342 |
| Seyoum | 39.5 | 8.2 | 10.3 | 10.3 | -α | 3.9 | 281 |
| Michael E et al | 49 | -α | 25 | 19.6 | -α | -α | 250 |
| Muluneh et al | 70.5 | -α | 19.7 | 11.6 | 13.3 | -α | 467 |

-α - Shows no reported data
