# Supplementary material for: Cervical cancer screening utilization and predictors among eligible women in Ethiopia: A systematic review and meta-analysis
Source: PLoS One. 2021 Nov 4;16(11):e0259339. doi: 10.1371/journal.pone.0259339 (PMC8568159; doi:10.1371/journal.pone.0259339)
Supplement: S3 Table — (DOCX) [file pone.0259339.s006.docx]

**S3 Table: Quality assessment of included studies**

| **Studies** | **Quality assessment criteria** | | | |
| --- | --- | --- | --- | --- |
|  | **Selection** | **Comparability** | **Outcome** | **Overall quality** |
| Shiferaw H et al (43) | **** | * | ** | 7 |
| Getachew S et al (44) | **** | * | ** | 7 |
| Bante SA et al (47) | **** | ** | ** | 8 |
| Aweke YH et al (56) | **** | * | ** | 7 |
| Nega AD et al (48) | **** | ** | ** | 8 |
| Nigussie T et al (49) | **** | ** | ** | 9 |
| Bayu H et al (64) | **** | ** | ** | 8 |
| Assefa AA et al (57) | **** | ** | ** | 8 |
| Gebreegziabher M et al (65) | **** | ** | ** | 8 |
| Solomon K et al (61) | **** | * | ** | 7 |
| Tefera and Mitiku (50) | **** | * | ** | 7 |
| Muluneh BA et al ([51](#_ENREF_51)) | **** | ** | ** | 8 |
| Seyoum T et al ([58](#_ENREF_58)) | **** | * | ** | 7 |
| Michael E et al ([42](#_ENREF_42)) | **** | * | ** | 7 |
| Galibo T et al ([41](#_ENREF_41)) | ***** | ** | ** | 9 |
| Kassa AS et al ([52](#_ENREF_52)) | **** | * | ** | 7 |
| Erku DA et al ([53](#_ENREF_53)) | **** | ** | ** | 8 |
| Woldetsadik AB ([45](#_ENREF_45)) | **** | * | ** | 7 |
| Aynalem BY et al ([54](#_ENREF_54)) | **** | * | ** | 7 |
| Asres T ([55](#_ENREF_55)) | *** | * | ** | 6 |
| Dulla D et al ([59](#_ENREF_59)) | *** | * | ** | 6 |
| Heyi WD et al ([62](#_ENREF_62)) | **** | ** | ** | 8 |
| Berhanu T et al ([66](#_ENREF_66)) | **** | ** | ** | 8 |
| Tekle T et al ([60](#_ENREF_60)) | *** | * | ** | 6 |
| Ashagrie A ([63](#_ENREF_63)) | *** | * | ** | 6 |

The Newcastle Ottawa Scale (NOS) was used **t**o assess the quality of included

** Two points, *** Three points; and **** four point
