# Supplementary material for: Cervical cancer screening utilization and predictors among eligible women in Ethiopia: A systematic review and meta-analysis
Source: PLoS One. 2021 Nov 4;16(11):e0259339. doi: 10.1371/journal.pone.0259339 (PMC8568159; doi:10.1371/journal.pone.0259339)
Supplement: S1 Appendix — (DOCX) [file pone.0259339.s007.docx]

**S1 Appendix: Specific searching detail in PubMed data base**

(((((("uterine cervical neoplasms"[MeSH Terms] OR ("uterine"[All Fields] AND "cervical"[All Fields] AND "neoplasms"[All Fields]) OR "uterine cervical neoplasms"[All Fields] OR ("cervical"[All Fields] AND "cancer"[All Fields]) OR "cervical cancer"[All Fields]) AND ("diagnosis"[Subheading] OR "diagnosis"[All Fields] OR "screening"[All Fields] OR "mass screening"[MeSH Terms] OR ("mass"[All Fields] AND "screening"[All Fields]) OR "mass screening"[All Fields] OR "screening"[All Fields] OR "early detection of cancer"[MeSH Terms] OR ("early"[All Fields] AND "detection"[All Fields] AND "cancer"[All Fields]) OR "early detection of cancer"[All Fields])) AND Uptake[All Fields]) OR uptake[All Fields]) OR ("statistics and numerical data"[Subheading] OR ("statistics"[All Fields] AND "numerical"[All Fields] AND "data"[All Fields]) OR "statistics and numerical data"[All Fields] OR "utilization"[All Fields])) AND (("women"[MeSH Terms] OR "women"[All Fields]) AND ("reproduction"[MeSH Terms] OR "reproduction"[All Fields] OR "reproductive"[All Fields]) AND ("Age"[Journal] OR "Age (Omaha)"[Journal] OR "Age (Dordr)"[Journal] OR "age"[All Fields]))) AND ("ethiopia"[MeSH Terms] OR "ethiopia"[All Fields]).
