# Supplementary material for: Cervical cancer screening utilization and predictors among eligible women in Ethiopia: A systematic review and meta-analysis
Source: PLoS One. 2021 Nov 4;16(11):e0259339. doi: 10.1371/journal.pone.0259339 (PMC8568159; doi:10.1371/journal.pone.0259339)
Supplement: Attachment — Submitted filename: CC meta-systematic analysis-Review comment.docx [file pone.0259339.s008.docx]

**Review comment**

**Cervical cancer screening utilization and predictors among reproductive-age women in Ethiopia: a systematic review and meta-analysis**

General comment: Very good and useful review that could provide evidence on barrier to CC screening in Ethiopia

Specific comments

IN the abstract as well as under background section, it is stated “Despite a remarkable progress in the reduction of maternal mortality, cervical cancer is the 73 second most commonly diagnosed cancer and the leading cause of cancer related death among 74 African women”. I agree with the reduction of MM. However, in view of the fact that CC is a major public health challenge and with its continued pressure, is it not naïve to argue about reduction of maternal mortality without showing how CC screening has contributed to that or contrary to this maintained MM in consequence higher.

Mention is made “The incidence, death rate and morbidities associated with cervical cancer is 79 significantly varies across the world; higher in the developing nations than compared to the 80 developed countries”(4), while again it is stated “The prevalence of cervical cancer screening is much higher at the Western countries than SSA” (11, 12); The two statements looks contradictory

Method

There is no clarity on whether this review is ‘systematic’ or ‘met-analysis’ or ‘both’. If it is both, it is imperative to clarify which part of it is systematic and meta-analysis OR clarify why both were considered.

From the statement “All published and unpublished studies through April 7, 2020” there were quite few concerns. Firstly what was the lower time frame. Could it be any document from time immemorial to April 7, 2020? Secondly, how were the unpublished reports captured? Were there any criterion set to identify those?

While WHO’s recommended age of CC screening is 30-50, it is not clear why in this study age 18-49 years was chosen? Is it not contradictory?

Results

The studies were drawn from five geographic regions: Addis Ababa, Amhara, Southern Nations, Nationalities and Peoples, Oromia and Tigray with only one from the national- level study. Couple of concern here. Firstly, how was the 18,067 women considered to estimate the pooled… proportionated in light of population size. Given the fact that studies are not proportional to the population of the regions, it would be difficult to reach conclusion as desired – this is about comparability of results. Secondly, I am wondering if this study could mirror realities for the country at large. Perhaps clarifying those and stating limitations may help.

I am not convinced that what are stated as ‘most common reasons that hinder the use of cervical cancer screening’ in the result section and conclusion section are aligned. This needs to be checked against the finding and corrected

Discussion

The fact that it was not clear on what of this review is meta and systematic, the discussion suffers much missing what is being discussed. Perhaps with clarification on how the two contributed to this review may help refine the discussion section
